# Fracture Resistance of CAD/CAM Lithium Disilicate and 3D-Printed Resin Crowns with Varying Occlusal Thickness: An In Vitro Study

**DOI:** 10.3390/ma19061180

**Published:** 2026-03-17

**Authors:** Bülent Kadir Tartuk, Eyyüp Altıntaş, Melike Şengül

**Affiliations:** 1 Department of Prosthodontics, Faculty of Dentistry, Bingöl University, Bingöl 12000, Türkiye; 2 Department of Prosthodontics, Faculty of Dentistry, Fırat University, Elazığ 23119, Türkiye; ealtintas@firat.edu.tr (E.A.); msengul@firat.edu.tr (M.Ş.)

**Keywords:** CAD/CAM crowns, 3D printing, additive manufacturing, conservative dentistry, occlusal thickness, dental materials

## Abstract

This in vitro study evaluated the fracture resistance of CAD/CAM-fabricated lithium disilicate and 3D-printed resin crowns with varying occlusal thicknesses (0.5, 1.0, and 1.5 mm) following thermomechanical aging. Sixty extracted human molars were assigned to six experimental groups (*n* = 10), categorized by crown material and occlusal thickness. The crowns were fabricated using CAD/CAM technology in accordance with the manufacturer’s protocol. All specimens underwent thermomechanical aging, which consisted of thermocycling between 5 and 50 °C (5500 cycles) combined with mechanical loading of 50 N at 1.6 Hz for 75,000 cycles. The fracture loads were measured using a universal testing machine, and the failure modes were assessed using scanning electron microscopy. Statistical evaluation was performed using two-way analysis of variance with Tukey’s post hoc test (α = 0.05). Both the material type and occlusal thickness had a statistically significant effect on fracture resistance (*p* < 0.001). Lithium disilicate crowns exhibited higher fracture loads than 3D-printed resin crowns independent of occlusal thickness. Although the fracture resistance of 3D-printed resin crowns was lower, specimens with occlusal thicknesses ≥1.0 mm exhibited fracture loads exceeding average physiological masticatory forces, suggesting that 3D-printed resin crowns may represent a clinically acceptable option for conservative posterior restorations. In contrast, crowns with an occlusal thickness of 0.5 mm demonstrated fracture resistance values below the reported functional masticatory loads. Additionally, the proportion of repairable fractures increased with increasing occlusal thickness for both materials. Overall, the findings suggest that an occlusal thickness of at least 1.0 mm may represent a reliable threshold for posterior restorations, whereas a thickness of 0.5 mm may be insufficient to withstand functional occlusal loads in molar regions.

## 1. Introduction

Recent advances in digital technologies have significantly transformed the field of dentistry, particularly in diagnostic procedures, treatment planning, and prosthetic manufacturing workflows [1]. Among these developments, the introduction of computer-aided design and computer-aided manufacturing (CAD/CAM) systems has enhanced clinical efficiency and precision while simultaneously accelerating the development and production of dental materials [2]. Currently, CAD/CAM technology is extensively applied in the fabrication of a wide range of restorations with superior esthetic properties, which are typically produced from industrially manufactured homogeneous blocks and shaped using subtractive milling techniques [3,4].

Among the wide range of CAD/CAM materials, lithium disilicate ceramic (LDC) blocks have become a popular choice, particularly for the fabrication of full-coverage crown restorations [5]. The literature consistently reports that lithium disilicate ceramics exhibit higher flexural strength and superior esthetic characteristics compared to leucite-reinforced ceramics and resin composite blocks [6]. Restorations fabricated using lithium disilicate have been shown to meet posterior load-bearing requirements with more conservative tooth preparation than conventional porcelain restorations [7].

However, despite these advantages, lithium disilicate ceramics also present certain limitations. Their relatively high stiffness and brittle behavior may increase the susceptibility to crack initiation and slow crack growth under cyclic loading conditions, which may ultimately result in catastrophic fracture [8,9]. In addition, ceramic restorations generally require sufficient material thickness to ensure mechanical reliability, which may limit their use in situations involving restricted occlusal space or when highly conservative tooth preparation is desired [10].

Although milling techniques enable the production of materials with high strength and low surface roughness, certain limitations remain, including microscopic crack formation and material wastage [11].

In recent years, three-dimensional (3D) printing technologies have been increasingly adopted in prosthetic dentistry, implant surgery, reconstruction, and orthodontics. These technologies offer several advantages, including lower material consumption and increased design flexibility, and they allow the fabrication of geometries with intricate features, such as undercuts or regions that are challenging to produce using subtractive manufacturing methods [12,13].

Light-curing composite resins which are used as restorative materials can also be fabricated using 3D printing. Compared with ceramic materials, composite resins exhibit lower hardness and elastic modulus, resulting in reduced wear of the opposing dentition, easier fabrication, and improved reparability. In addition, these materials offer advantages such as easier fabrication, improved marginal adaptation, and the possibility of repair in cases of minor fracture [14,15]. The mechanical behavior of 3D-printed resin materials is also influenced by their layer-by-layer manufacturing process, which may introduce variations in interlayer bonding and polymerization. These microstructural characteristics may affect fracture resistance and crack propagation patterns [16]. Despite this growing interest in the clinical application of 3D-printed CAD/CAM materials, the existing literature indicates that their clinical performance has not been adequately supported by a limited number of studies [17].

In parallel with these technological advancements, contemporary restorative dentistry has increasingly emphasized minimally invasive treatment concepts. The preservation of the remaining tooth structure is a fundamental concept in tooth preparation and is closely associated with the fracture resistance of restorations. Restorations with reduced thicknesses provide a viable clinical option for patients with restricted occlusal space and younger individuals, as they help prevent pulp exposure. Earlier investigations have demonstrated that hybrid ceramic crowns with reduced thicknesses can sustain fracture loads compatible with posterior function [18,19]. Depending on the restorative material, reported occlusal thickness values for crowns have been shown to vary from 0.3 to 2.0 mm [20,21,22].

The evaluation of the fracture resistance of CAD/CAM molar crowns with reduced occlusal thickness is essential for preserving tooth structure and enhancing restoration durability [23]. However, evidence regarding the optimal thickness of restorations fabricated using 3D printing remains limited. Further investigations are required to clarify the mechanical and physical properties of CAD/CAM materials produced using contemporary prosthetic manufacturing techniques. In particular, data on the influence of the material type and thickness on the fracture resistance of molar crowns are scarce. Although previous studies have primarily focused on lithium disilicate restorations, information on the optimal thickness of 3D-printed resin crowns remains insufficient.

Accordingly, the present study aimed to evaluate and compare the fracture resistance of CAD/CAM-fabricated lithium disilicate and 3D-printed resin crowns with different occlusal thicknesses.

The null hypothesis (H_0_) of this study was that material type and occlusal thickness would have no statistically significant effect on the fracture resistance of the tested crowns.

## 2. Materials and Methods

### 2.1. Study Design

The sample size calculation was performed using the G*Power statistical software (version 3.0.10; Franz Faul, Kiel University, Kiel, Germany). The primary outcome variable was fracture resistance (N). A two-way ANOVA model incorporating two independent factors was applied for the sample size calculation: material type (lithium disilicate and 3D-printed resin) and occlusal thickness (0.5, 1.0, and 1.5 mm).

**Figure 1:**
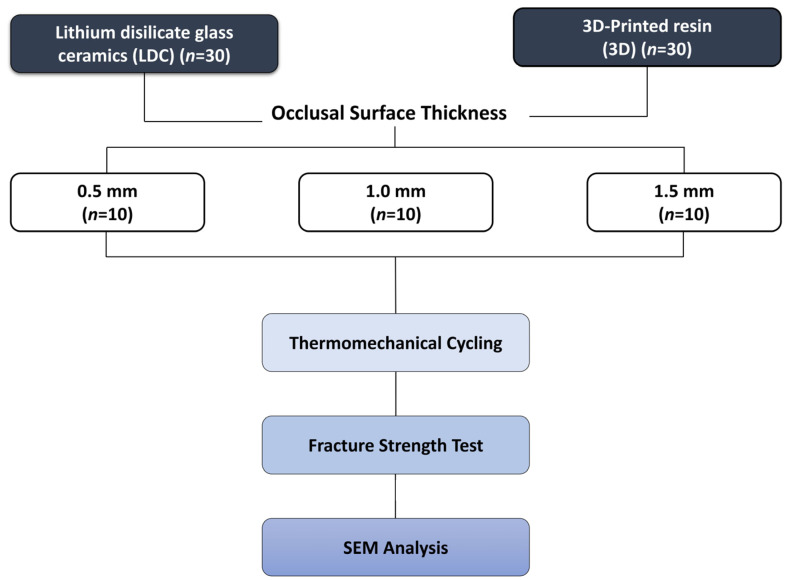
Schematic illustrating the experimental process.

A large effect size (f = 0.40) was assumed based on previous in vitro studies that evaluated the fracture resistance of CAD/CAM restorations with different materials and occlusal thicknesses. The significance level was set at α = 0.05, and the statistical power was defined as 1 − β = 0.80. Under these assumptions, a sample size of 10 specimens per group was calculated. Accordingly, a total of 60 specimens were included in the study to provide sufficient statistical power for the evaluation of fracture resistance (Figure 1).

This in vitro study was conducted using extracted human molars obtained for periodontal reasons from healthy individuals aged between 18 and 55 years. Teeth were collected according to a protocol approved by the local ethics committee (Approval No: 2025-12/1). All specimens were examined under 5× magnification, and only intact teeth that were free of caries, cracks, or structural defects were included. Teeth with similar buccolingual and mesiodistal dimensions were selected, and crown dimensions were measured using a digital caliper (Absolute Digimatic Caliper; Starrett Inc., Athol, MA, USA). Specimens that deviated by more than 10% from the mean values were excluded. Calculus deposits and surface stains were removed using manual instruments (Aesculap Dental, Tuttlingen, Germany). The teeth were stored at room temperature in a 0.9% saline solution containing 0.1% thymol until use.

### 2.2. Tooth Preparation

**Figure 2:**
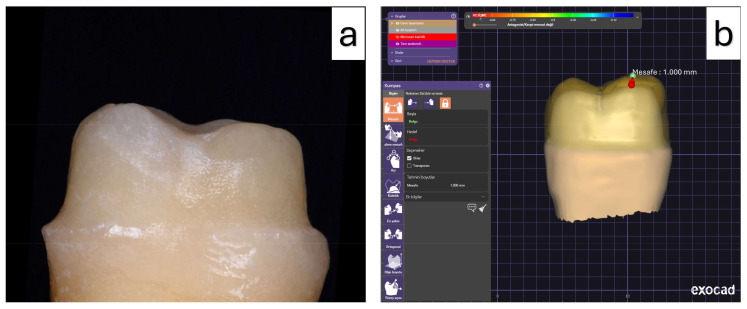
(**a**) Tooth prepared with standardized anatomical occlusal reduction according to the study protocol. (**b**) Digital model generated using CAD software for evaluation of occlusal thickness and spatial distance measurements.

All tooth preparations were performed by an experienced prosthodontist under water cooling using diamond rotary burs (medium-grit, rounded-end tapered diamond burr; Brasseler USA, Savannah, GA, USA). To ensure standardization, a new bur was used after each preparation. Each tooth received standardized preparation consisting of approximately 1.0 mm occlusal reduction and 0.8 mm circumferential reduction, with a 0.5 mm chamfer finish line and a total occlusal convergence angle ranging between 6° and 8°. Undercuts were avoided during the preparation (Figure 2).

The extent of tooth reduction was verified using the silicone index obtained prior to preparation. The internal line angles were refined using a yellow-coded cylindrical bur (fine-grit cylindrical diamond bur; Brasseler USA, Savannah, GA, USA), and the prepared surfaces were subsequently polished with polishing discs (OptiDisc; Kerr Corporation, Orange, CA, USA) to enhance restorative adaptation and achieve a more uniform cement thickness.

To ensure standardization of tooth preparations, all preparations were performed by a single experienced operator using a standardized preparation protocol. Depth-cutting burs were used to guide occlusal reduction, and the preparation dimensions were periodically verified with a digital caliper to maintain the intended reduction. This approach was adopted to minimize variability and ensure uniformity among the specimens.

### 2.3. Restoration Fabrication

The materials evaluated in this study are summarized in Table 1. For restoration fabrication, the teeth in both material groups were randomly allocated into three subgroups according to occlusal thickness. All crowns were designed and manufactured using the CAD/CAM workflow. Initially, the prepared teeth were digitized using an intraoral scanner (TRIOS 4; 3Shape, Copenhagen, Denmark), and full-coverage crown restorations were designed using CAD software (Exocad v3.0; Exocad GmbH, Darmstadt, Germany).

**Figure 3:**
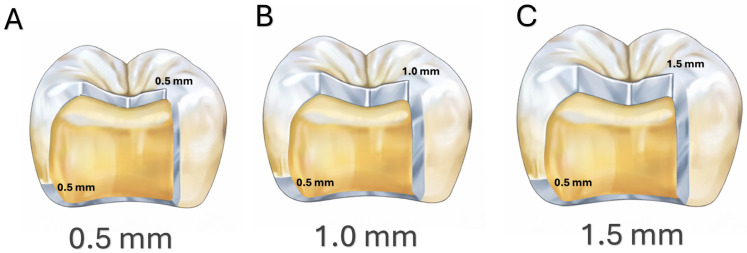
Cross-sectional schematic representation of full-coverage crown designs; with (**A**) 0.5 mm occlusal thicknesses, (**B**) 1.0 mm occlusal thicknesses, and (**C**) 1.5 mm occlusal thicknesses used in this study.

Occlusal thickness was defined digitally by virtually increasing or decreasing the occlusal surface in the fissure area. The restorations wall and margin thicknesses were kept uniform across all specimens, while occlusal thicknesses were set at 0.5 mm, 1.0 mm, and 1.5 mm. A uniform cement space of 50 µm was applied to all restorations. The same digital design files were used to fabricate both lithium disilicate and 3D-printed resin crowns (Figure 3).

The lithium disilicate restorations were fabricated using a CAM system (DWX-52D; Roland DG Corporation, Hamamatsu, Japan). After milling, the restorations were crystallized in a ceramic furnace (Vario Press 300.e; Zubler USA Inc., Hicksville, NY, USA) at 880 °C for 30 min. Following crystallization, the restorations were polished using rubber polishing wheels, and an additional glazing procedure was performed at 700 °C. All ceramic restorations were polished using a polishing set (OptraDisc; Ivoclar Vivadent, Schaan, Liechtenstein).

The 3D-printed crowns were fabricated using a stereolithography (SLA) printer (Form 3; Formlabs Inc., Somerville, MA, USA) with a layer thickness of 50 µm according to the manufacturer’s recommendations. The printing speed was automatically optimized by the printer’s software (PreForm, version 3.54.1; Formlabs Inc., Somerville, MA, USA) based on the material’s specific curing requirements. Following fabrication, the specimens were rinsed in 95% isopropyl alcohol for 3 min using a Form Wash unit, after which the support structures were carefully detached. Subsequently, post-curing was performed in a Form Cure unit at 60 °C for 20 min. Prior to cementation, the intaglio surfaces of all restorations were conditioned by airborne-particle abrasion using 50 µm aluminum oxide (Al_2_O_3_) at 2 bars for 10 s.

### 2.4. Cementation Procedure

After ultrasonic cleaning, the internal surfaces of the lithium disilicate glass-ceramic restorations were etched with 5% hydrofluoric acid (Ceramic Etchant; VOCO GmbH, Cuxhaven, Germany) for 20 s. The surfaces were then rinsed with water for 60 s and air-dried. Subsequently, a silane coupling agent (BIS-SILANE; Bisco Inc., Schaumburg, IL, USA) was applied, followed by the application of a universal bonding primer (Adhese Universal; Ivoclar Vivadent AG, Schaan, Liechtenstein). After a dwell time of 60 s, the surfaces were gently air-dried.

For 3D-printed resin restorations, surface pretreatment of the intaglio areas was performed in accordance with the manufacturer’s recommendations. The internal surfaces were first subjected to airborne-particle abrasion using 50 µm aluminum oxide particles applied with an air-abrasion device (Basic Quattro; Renfert GmbH, Hilzingen, Germany). The abrasion procedure was carried out at an air pressure of 0.15 MPa. Subsequently, a universal bonding primer was applied, and the surface was gently air-dried without light activation.

The tooth surfaces were etched with 37% phosphoric acid gel (Ultra-Etch; Ultradent Products Inc., South Jordan, UT, USA) for 30 s on enamel and 15 s on dentin, rinsed with water, and gently air-dried. A universal adhesive (Scotchbond Universal Plus; 3M Oral Care, St. Paul, MN, USA) was subsequently applied, allowed to react for 30 s, and air-dried.

All restorations were cemented using a dual-cure resin cement (G-CEM LinkForce; GC Corporation, Tokyo, Japan). During cementation, each crown was seated on the prepared tooth under a constant load of 20 N using a digital force gauge (FG-3005; Lutron Electronic Enterprise Co., Taipei, Taiwan) to standardize seating pressure. After placement, preliminary light polymerization was performed for 3 s on each surface to facilitate the removal of excess cement. Final polymerization was completed by light-curing each surface for 20 s using an LED curing unit (VALO Cordless; Ultradent Products Inc., South Jordan, UT, USA) with a light intensity of 1200 mW/cm^2^.

To enhance the clinical relevance of the experimental setup, simulation of the periodontal ligament and alveolar bone were performed. The root surfaces were coated with a 0.2–0.3 mm layer of vinyl polysiloxane-based elastomeric material (Elite HD+; Zhermack, Badia Polesine, Italy) to simulate periodontal ligament compliance. The teeth were subsequently embedded in polyvinyl resin.

### 2.5. Thermomechanical Aging

To simulate intraoral conditions and approximate the clinical environment, all specimens underwent a standardized thermomechanical aging protocol. The aging process was conducted using a chewing simulator incorporating an integrated thermocycling module (CS-4.8 Professional Line; SD Mechatronik GmbH, Feldkirchen-Westerham, Germany).

Thermal cycling and mechanical loading were applied concurrently to replicate the functional occlusal forces and temperature fluctuations encountered in the oral cavity. Vertical mechanical loading was applied with a magnitude of 50 N at a frequency of 1.6 Hz for a total of 75,000 cycles, which corresponds to approximately six months to one year of clinical masticatory activity according to previous studies [24].

Thermal cycling was performed within the same experimental setup by repeatedly immersing the specimens in water baths maintained at 5 °C and 50 °C. Each specimen was held at the target temperature for 20 s, with an intermediate transfer time of 2 s between the baths, resulting in a total of 5500 thermal cycles. This protocol allowed the specimens to be exposed to simultaneous thermal variations and mechanical loading under standardized laboratory conditions.

### 2.6. Fracture Resistance Test and Failure Mode Analysis

After the thermomechanical aging procedure, the tested specimens were subjected to fracture resistance testing using a universal testing machine (Shimadzu Model AGS-X; Shimadzu Corporation, Kyoto, Japan). The crowns were tested while adhering to teeth embedded in acrylic resin blocks. The occlusal surface was centrally loaded using a steel spherical indenter with a diameter of 5 mm under compressive conditions.

**Figure 4:**
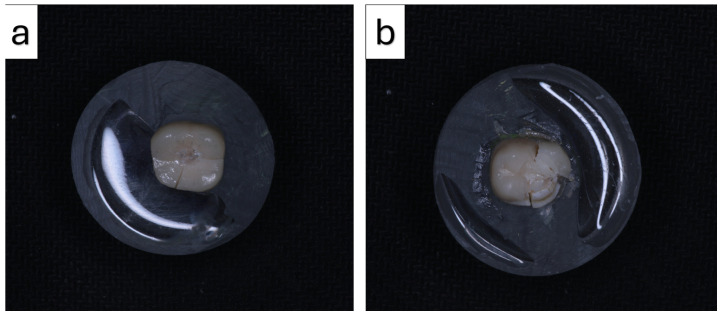
Representative the fracture patterns observed in the specimens. (**a**) Repairable failure and (**b**) non-repairable failure characterized.

The load was applied vertically at a crosshead speed of 0.5 mm/min until the initial crack formation or complete fracture occurred. The maximum load at failure was recorded in Newtons (N) for each specimen (Figure 4).

**Figure 5:**
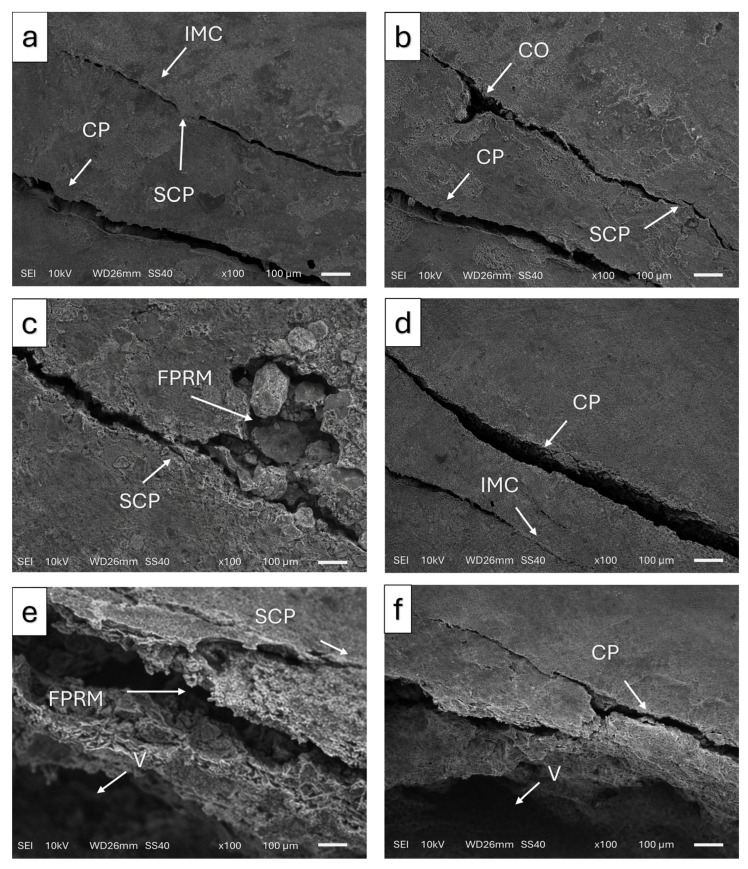
Representative SEM images of the fractured surface of each group. (**a**) 3D-1.5 mm, (**b**) LDC-1.5 mm, (**c**) 3D-1.0 mm, (**d**) LDC-1.0 mm, (**e**) 3D-0.5 mm, (**f**) LDC-0.5 mm. CO: Crack origin, CP: Crack propagation, FPRM: Filler particles regarding matrix, IMC: Internal micro crack, SCP: Stopped crack propagation, V: Void.

Following fracture testing, the fractured specimens were further analyzed using scanning electron microscopy (SEM; JEOL JSM-6510, JEOL Ltd., Tokyo, Japan) (Figure 5). Prior to examination, the specimens were sputter-coated with a thin gold layer using a sputter coater (Emitech K550, Quorum Technologies Ltd., Lewes, UK) to enhance surface conductivity. SEM observations were performed under high-vacuum conditions at an accelerating voltage of 10 kV. The failure modes were categorized as follows:

- Repairable failures (R): Fractures limited to the restorative material without involvement of the underlying tooth structure, such as cohesive chipping within the crown material or adhesive failure with a detached fragment. In these cases, the restoration may be repaired or replaced without damaging the supporting tooth tissues.
- Non-repairable failures (NR): Catastrophic fractures extending into the underlying tooth structure or below the simulated bone level, including crown-root fractures that result in substantial structural loss and render the restoration non-repairable.

### 2.7. Statistical Analysis

All statistical procedures were carried out using statistics software (IBM SPSS; version 20.0; IBM Corp., Armonk, NY, USA). Data normality was assessed with the Shapiro–Wilk test, while variance homogeneity was evaluated using Levene’s test. The results confirmed that the data met the requirements for parametric analysis (*p* > 0.05).

To evaluate the effects of restorative material type (lithium disilicate and 3D-printed resin) and occlusal thickness (0.5, 1.0, and 1.5 mm) on fracture resistance, a two-way analysis of variance (ANOVA) was performed. This approach enabled the evaluation of not only the main effects of each factor but also the potential interaction between material type and thickness. When statistically significant differences were observed, post hoc multiple comparisons were performed using Tukey’s honestly significant difference (HSD) test. The threshold for statistical significance was set at *p* < 0.05.

To provide an additional clinically meaningful interpretation of the differences in fracture resistance between occlusal thickness groups, percentage change values were calculated based on the mean fracture load values using the formula [(Mean_B − Mean_A)/Mean_A] × 100. These percentage values were used solely for descriptive purposes to illustrate the relative magnitude of differences between groups. All statistical analyses, including significance testing, were conducted using the absolute fracture load values (N).

Differences in the failure mode distribution (repairable vs. non-repairable) between materials at each occlusal thickness level were analyzed using Fisher’s exact test to assess the clinical relevance and repairability of the restorations. The level of statistical significance was set at *p* < 0.05.

## 3. Results

**Figure 6:**
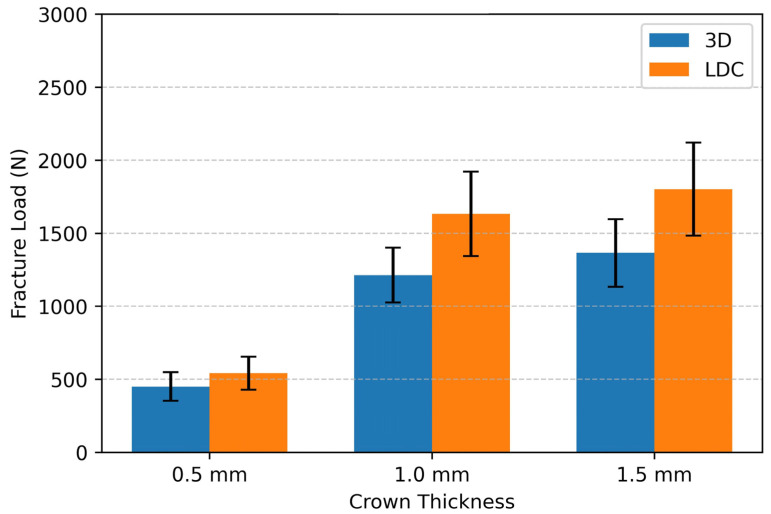
Column chart presents mean fracture load values (N) with standard deviation for lithium disilicate (LDC) and 3D-printed resin (3D) crowns with different occlusal thicknesses (0.5, 1.0 and 1.5 mm).

The mean fracture resistance values and standard deviations for both materials at different occlusal thicknesses are summarized in Table 2. The highest mean fracture load was observed in the lithium disilicate crown group with an occlusal thickness of 1.5 mm (1802 ± 318 N), whereas the lowest mean fracture load was recorded in the 3D-printed resin crown group with an occlusal thickness of 0.5 mm (451 ± 98 N) (Figure 6).

Two-way ANOVA revealed that both material type (F (1, 54) = 82.60, *p* < 0.001, ηp^2^ = 0.60) and occlusal thickness (F (2, 54) = 109.40, *p* < 0.001, ηp^2^ = 0.80) had a statistically significant effect on fracture resistance. In addition, a significant interaction between the material type and occlusal thickness was observed (F (2, 54) = 7.20, *p* < 0.001, ηp^2^ = 0.21) (Table 3).

An increase in occlusal thickness resulted in a significant increase in fracture resistance in both the material groups. Pairwise comparisons demonstrated that lithium disilicate crowns exhibited significantly higher fracture resistance than 3D-printed resin crowns of all thicknesses (*p* < 0.05). The mean differences between LDC and 3D-printed resin crowns were 91 N at 0.5 mm thickness, 420 N at 1.0 mm thickness, and 437 N at 1.5 mm thickness. The 95% confidence intervals for the differences were −49 to 231 N for the 0.5 mm thickness group, 280 to 560 N for the 1.0 mm thickness group, and 297 to 577 N for the 1.5 mm thickness group (Table 4).

When the percentage changes between the thickness groups were analyzed, increasing the occlusal thickness from 0.5 mm to 1.0 mm resulted in an increase of 201.48% in the mean fracture resistance for the lithium disilicate group and 169.18% for the 3D-printed resin group. Similarly, increasing the occlusal thickness from 0.5 mm to 1.5 mm led to increases of 232.47% and 202.66% in the lithium disilicate and 3D-printed resin crowns, respectively. Statistical significance (*p* < 0.05) was determined based on absolute fracture load differences (N), not percentage values. Confidence intervals refer to the absolute mean difference in the fracture load values (N). No statistically significant differences were observed between the 1.0 mm and 1.5 mm thickness groups for either material (*p* > 0.05) (Table 5).

**Figure 7:**
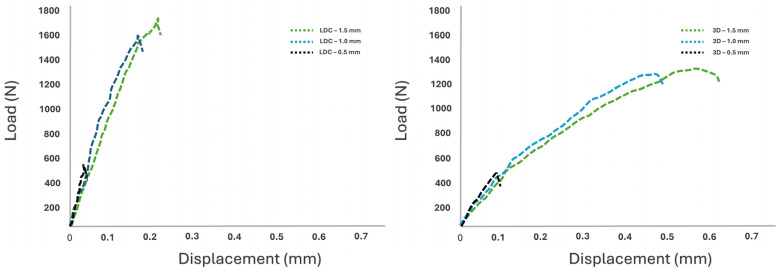
Representative load–displacement curves obtained during fracture testing of lithium disilicate (LDC) and 3D-printed resin crowns (3D) with different occlusal thicknesses (0.5, 1.0, and 1.5 mm) after thermomechanical aging.

Representative force–displacement curves (Figure 7) further demonstrate the variations in the mechanical response of the materials evaluated in this study. In both material groups, an increase in occlusal thickness was associated with higher fracture loads and greater displacement prior to failure. 3D-printed resin crowns, particularly at occlusal thicknesses of 1.0 mm and 1.5 mm, showed a more progressive load–displacement profile and a higher deformation capacity before fracture. In contrast, crowns with an occlusal thickness of 0.5 mm in both material groups were characterized by earlier failure and reduced displacement.

**Figure 8:**
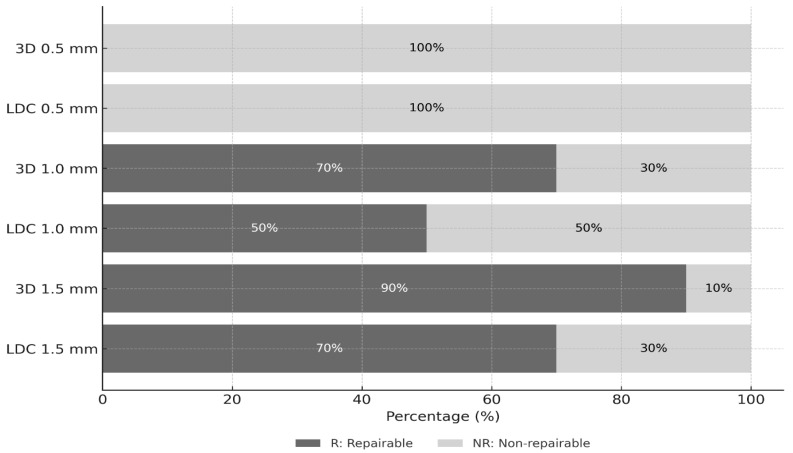
Percentage distribution of failure modes (repairable vs. non-repairable) for lithium disilicate (LDC) and 3D-printed resin (3D) crowns with different occlusal thicknesses (0.5, 1.0 and 1.5 mm).

Regarding failure modes, all specimens with an occlusal thickness of 0.5 mm exhibited non-repairable (NR) fractures. In contrast, the proportion of repairable (R) fractures increased with increasing occlusal thickness in both groups (Table 6). In the 3D-printed resin group, 90% of the specimens with a thickness of 1.5 mm demonstrated repairable fracture patterns, whereas this proportion was 70% in the lithium disilicate group (Figure 8).

At occlusal thicknesses of 1.0 mm and 1.5 mm, the failure mode distributions did not differ significantly between the materials (Fisher’s exact test; *p* = 0.65 and *p* = 0.58, respectively). At a thickness of 0.5 mm, all specimens exhibited non-repairable failures; therefore, statistical comparison was not applicable.

## 4. Discussion

The results of the present investigation revealed distinct differences in fracture resistance between CAD/CAM-fabricated lithium disilicate ceramic crowns and 3D-printed resin-based crowns with varying occlusal thicknesses (0.5, 1.0, and 1.5 mm). The results of the two-way analysis of variance indicated that both material type and occlusal thickness exerted a statistically significant effect on fracture resistance, with a significant interaction observed between these two factors (*p* < 0.001). Consequently, the null hypothesis was rejected. Earlier investigations have similarly demonstrated the influence of restoration thickness as an important factor influencing mechanical performance [14,25]. An increase in occlusal thickness was associated with a significant improvement in fracture resistance for both materials; however, this effect appeared to be more pronounced in the lithium disilicate group.

Moreover, the significant interaction between material type and thickness suggests that material selection should not be based solely on intrinsic mechanical properties but should also consider the planned occlusal thickness of the restoration.

Although the use of natural teeth is generally preferred in laboratory studies to better approximate clinical conditions, substantial variations in tooth anatomy, patient age, and post-extraction storage can complicate standardization [26]. Nevertheless, in vitro studies aimed at evaluating the preparation design and restorative performance should closely replicate clinical scenarios. Consequently, the use of highly simplified geometries or metallic dies with flat occlusal surfaces may not accurately reflect intraoral loading conditions [18]. In this study, extracted human teeth were selected to achieve a more clinically relevant experimental model.

To ensure consistency among the specimens, the silicone index technique was employed during tooth preparation to maintain standardized preparation geometry and dimensions. An alternative approach would have been to reduce tooth dimensions according to the planned restoration thickness. However, this strategy could have adversely affected biomechanical outcomes owing to increased tooth structure loss and reduced substrate support. In addition, because hoop stress is directly related to tooth diameter, this approach might have introduced an additional confounding variable among the experimental groups [25].

Although resin cements act as stress-absorbing interlayer between the crown and tooth structure, it has been reported that increases in cement thickness can influence occlusal load distribution and fracture behavior [27]. In CAD/CAM workflows, a cement space of approximately 50 µm is recommended to optimize seating and mechanical performance [28]. In the present study, parameters such as cement layer thickness were standardized using identical CAD design settings across all specimens to eliminate potential confounding effects related to cementation.

Thermomechanical aging was performed prior to fracture testing to simulate the combined mechanical and thermal challenges encountered under clinical conditions. Previous studies have demonstrated that thermomechanical aging procedures can significantly reduce the fracture load of restorative materials [29,30].

Additionally, aging protocols are important for evaluating the fracture behavior of restorations under simulated clinical service conditions. Repetitive occlusal loading promotes fatigue-related damage, and failure modes may evolve over time depending on loading conditions, material properties, and restoration geometry. In crown restorations, both chemically assisted slow crack growth and mechanical fatigue contribute to crack initiation and propagation, during which microscopic cracks may progressively develop within the material [31,32].

Although dynamic fatigue tests offer valuable information regarding the behavior of dental materials under cyclic loading, static axial loading tests remain a fundamental starting point for evaluating fracture resistance [33]. Such tests not only allow the assessment of the inherent strength of restorative materials but also provide meaningful data for optimizing restoration geometry and supporting clinical decision-making processes [22].

Contemporary restorative strategies increasingly favor minimally invasive concepts, thereby promoting the use of thinner restorations [34]. Such restorations are frequently preferred in clinical practice, because maximal preservation of tooth structure supports pulp vitality and reduces the risk of postoperative sensitivity [14]. Previous studies have indicated that maintaining a minimum dentin thickness of at least 2 mm after preparation represents a critical biological threshold [35]. Furthermore, crowns designed with minimal occlusal thickness have been highlighted as effective treatment options in cases of severe tooth wear and erosion [36].

Within this context, the occlusal thickness values investigated in this study (0.5, 1.0, and 1.5 mm) were selected to represent different clinical preparation approaches in posterior restorations. A thickness of 1.5 mm represents a more conventional crown design that is frequently used to ensure sufficient mechanical strength in posterior load-bearing areas. The 1.0 mm thickness corresponds to the commonly recommended minimum occlusal thickness for monolithic CAD/CAM crowns in posterior regions [37]. In contrast, the 0.5 mm thickness reflects an ultrathin restorative design associated with minimally invasive restorative concepts and conservative tooth preparation approaches [38].

However, a reduction in occlusal ceramic thickness may compromise the structural integrity, mechanical resistance, and long-term clinical performance of restorations [39]. Therefore, evaluating the fracture resistance of CAD/CAM-fabricated molar crowns with reduced occlusal thickness is of considerable clinical relevance as it provides insight into material durability and long-term performance under functional loading conditions [40]. In this context, occlusal thicknesses of 0.5 mm and 1.0 mm were intentionally included in the present study, despite being below the manufacturer-recommended limits, to represent minimally invasive restorative strategies.

The present findings revealed that restorations with an occlusal thickness of 0.5 mm exhibited significantly lower mean fracture resistance than those with a thickness of 1.0 mm. Increasing the material thickness resulted in a progressive improvement in the fracture resistance of both material groups. In particular, crowns with an occlusal thickness of 0.5 mm demonstrated fracture resistance values below average masticatory forces and were therefore considered clinically insufficient [41]. In contrast, restorations with an occlusal thickness of ≥1.0 mm exhibited mechanically acceptable performance. These results are consistent with previous reports that discouraged the use of restorations thinner than 1.0 mm in patients exposed to high occlusal loads [36,42].

In contrast to the present findings, Essam et al. [43] suggested that lithium disilicate crowns with a thickness of 0.5 mm may still be clinically applicable. The discrepancies between studies were attributed to differences in experimental parameters, particularly the lack of methodological standardization. Such variations highlight the importance of standardized testing protocols when evaluating the mechanical performance of reduced-thickness restorations.

Similarly, Bergamo et al. [37] investigated crowns with thicknesses of 0.5 mm, 1.0 mm, and 1.5 mm and reported no significant difference in survival between the 1.0 mm and 1.5 mm groups, with both exhibiting comparable clinical performance. These observations support the findings of the present study, suggesting that occlusal thickness of 1.0 mm may be considered a clinically safe lower threshold. Conversely, the reduced survival reported for 0.5 mm-thick restorations indicates limited clinical applicability at this thickness level.

Reducing the thickness of ceramic restorations has been suggested to increase the stress concentration at the cement interface, which may contribute to a reduction in overall fracture resistance [44]. This phenomenon explains the inferior mechanical performance observed in ultrathin restorations and underscores the importance of maintaining minimum occlusal thickness to ensure structural stability.

Park et al. [34] reported a decrease in fracture resistance with an increase in thickness of the crowns fabricated using 3D printing technology. They attributed this observation to the polymer chemistry of the material, suggesting that although the oligomers formed exhibit a relatively high molecular weight and intrinsic mechanical strength, a low cross-linking density combined with a high deformation capacity may increase material flexibility and negatively affect fracture resistance. Such discrepancies among studies are likely attributable to methodological differences and a lack of standardization across experimental designs. In this regard, experimental models employing metallic dies may exhibit stress distribution characteristics that differ from those observed in natural tooth-supported restoration systems.

In the present study, the incorporation of BisEMA into the resin matrix was associated with a statistically significant increase in the fracture toughness of 3D-printed crowns with an occlusal thickness of ≥1.0 mm (*p* < 0.05). Conversely, specimens with a thickness of 0.5 mm exhibited a marked reduction in fracture resistance, likely due to a combination of lower surface hardness, increased wear susceptibility, and insufficient structural thickness.

The relatively low elastic modulus of 3D-printed resin materials, along with interlayer bonding deficiencies and potential polymerization inconsistencies, may adversely affect long-term restoration integrity and marginal stability under clinical conditions. In layer-by-layer additive manufacturing processes, weak interfacial adhesion between successive layers, particularly in the occlusal region, may compromise mechanical performance and contribute to premature failure [34]. Therefore, determining the appropriate occlusal thickness for crowns fabricated using 3D printing technology is critical importance. Furthermore, future material development strategies, such as the incorporation of reinforcing filler particles, may enhance the physical and mechanical properties of resin matrices and improve the clinical viability of these materials for definitive prosthetic restorations [45,46].

Fracture resistance consistently favored lithium disilicate crowns over 3D-printed resin crowns across the tested thickness range. Lithium disilicate ceramics (IPS e.max) are characterized by high rigidity and stiffness and possess a markedly higher elastic modulus than both natural tooth structures and resin-based crowns. These material characteristics are associated with increased load-bearing capacity and fracture resistance. In addition, adequate bonding within the ceramic–cement–tooth complex has been reported to significantly improve the mechanical performance of ceramic restorations [47]. In glass-ceramic systems, both chemical bonding to the inorganic phase and appropriate surface treatments play a critical role. Specifically, hydrofluoric acid etching followed by silane applications have been shown to enhance bonding effectiveness in silica-based glass-ceramic restorations [48].

The evaluation of failure modes also provides clinically relevant information regarding the potential management of fractured restorations. Repairable failures, such as minor chipping or localized fractures, may allow intraoral repair and continued clinical use of the restoration. In contrast, non-repairable failures involving catastrophic fractures typically require the complete replacement of the restoration [49]. Replacing the entire restoration may not be the most practical solution. Not only is the replacement time consuming and costly, but there is also the risk of damaging the prepared abutment while attempting to remove the restoration [50]. Therefore, the analysis of failure patterns may offer additional insight into the clinical behavior of restorative materials beyond fracture resistance values alone.

The failure patterns observed in the 3D-printed resin groups were predominantly characterized by diffuse crack formation within the tooth–restoration complex without complete debonding or separation of the occlusal covering. This behavior may be attributed to the relatively low elastic modulus of 3D-printed occlusal restorations, which allows for greater energy absorption and increased flexibility under functional loading conditions [51]. Such failure characteristics are considered favorable within the framework of minimally invasive dentistry, as failures that do not compromise the underlying tooth structure may facilitate replacement and contribute to the improved longevity and prognosis of the restored tooth.

SEM analyses performed after the fracture test revealed internal microcracks, porosities, and microstructural defects, particularly in the 3D-printed resins. After thermal cycling, these defects became more pronounced, with increased crack formation on the fracture surfaces. This phenomenon may be attributed to water absorption and the accumulation of internal stresses during repeated thermal expansion and contraction, which can facilitate crack initiation and propagation in 3D-printed resins [52].

In contrast, the lithium disilicate (LDC) group predominantly exhibited catastrophic fracture patterns. This finding is likely related to the high elastic modulus of glass-ceramic materials, which limits their ability to dissipate stresses and promote rapid crack propagation once fracture initiation occurs [53]. Owing to their limited capacity for elastic deformation, ceramic materials are unable to effectively absorb applied stresses, resulting in abrupt and irreversible fractures [54].

In the present study, crowns with an occlusal thickness of 1.5 mm exhibited a higher proportion of repairable failure. This finding may be explained by the tendency of crack propagation to remain confined within the restorative material as the thickness increases. Ko et al. [55], reported a strong correlation between fractured energy and structural thickness, indicating that thicker restorative structures are better able to absorb and dissipate applied stresses before catastrophic failure occurs.

Similarly, previous studies have shown that restoration thickness significantly influences survival outcomes. In one such study, lithium disilicate occlusal veneers with varying thicknesses (0.5, 0.8, and 1.2 mm) were evaluated, and thicker restorations exhibited significantly higher survival rates than their thinner counterparts [56]. These findings support the observation that increased thickness contributes to improved mechanical reliability.

In contrast, Corbani et al. [38], reported a higher incidence of non-repairable failures in crowns with a thickness of 1.5 mm compared to those with a thickness of 1.0 mm, while all failures observed in 0.5 mm-thick crowns were classified as repairable. The authors attributed these differences to the use of SLA-fabricated abutment dies instead of natural teeth, suggesting that discrepancies in the elastic modulus between the supporting substrates may lead to variations in the stress distribution and fracture behavior. This highlights the importance of using clinically relevant substrates when interpreting failure patterns.

Our findings have several important clinical implications. Targeting an occlusal thickness of 1.0 mm rather than 1.5 mm during tooth preparation may help preserve sound tooth structure without compromising mechanical performance. In other words, a thickness of 1.0 mm appears to offer an optimal balance between minimal invasiveness and sufficient fracture resistance. This approach aligns with the principles of minimally invasive dentistry and is particularly relevant in younger patients or in cases where pulpal proximity is a concern [57].

Despite the valuable insights provided by this study, its findings should be interpreted within the context of certain limitations. As with all in vitro investigations, laboratory conditions cannot fully replicate the complexity of the oral environment [58]. One of the primary limitations of the present study was that fracture resistance was evaluated under static vertical loading only. In clinical settings, restorations are exposed to a combination of vertical and lateral forces during mastication, which may influence fracture behavior differently. Therefore, the results should be interpreted with caution when extrapolating to clinical situations, and further long-term clinical studies are needed to validate these findings.

Furthermore, although careful specimen selection was performed, variations in the morphological characteristics and hydroxyapatite composition of the supporting teeth may have affected standardization. Moreover, while the fracture resistance outcomes of this in vitro study cannot be regarded as direct indicators of long-term clinical performance, they offer valuable comparative insight into how material type and occlusal thickness affect mechanical behavior.

Finally, additional studies are warranted to investigate the effects of storage conditions, aging duration, and temperature variation on the long-term performance of CAD/CAM ceramic restorations. Future research incorporating dynamic loading, multidirectional forces, and long-term clinical evaluation would contribute to a more comprehensive understanding of their clinical behavior.

## 5. Conclusions

Within the limitations of this in vitro study, all material groups with occlusal thicknesses of 1.0 mm and 1.5 mm demonstrated fracture resistance values that were considered acceptable for potential clinical applications. In contrast, CAD/CAM crowns designed with reduced occlusal thicknesses (0.5 mm) showed limited fracture resistance, suggesting insufficient reliability for molar applications.

The findings of the present study suggest that occlusal thickness plays an important role in influencing both fracture resistance and failure mode. Across all the evaluated thickness levels, lithium disilicate crowns exhibited higher fracture resistance than those of 3D-printed resin crowns.

From a clinical perspective, an occlusal thickness of 1.0 mm may represent a promising threshold for balancing mechanical reliability and conservative tooth preparation in posterior restorations. However, this value should be interpreted as an in vitro benchmark rather than a definitive universal clinical recommendation, and further clinical studies are required to confirm these findings.

## Figures and Tables

**Table 1:** Chemical composition of the materials tested.

Material	Product Name	Composition	Manufacturer
Lithium disilicate glass ceramics (LDC)	IPS e.max CAD^®^	Silicon dioxide: 57.0–<80.0% Lithium oxide: 11.0–<19.0% Potassium oxide: 0.0–<13.0% Other oxides: 1–<8%	Ivoclar Vivadent AG, Schaan, Liechtenstein
3D-Printed Resin (3D)	Crowntec^®^	BisEMA (Bisphenol A polyethylene glycol diether dimethacrylate): 50–<75% Methyl benzoylformate: 1–<5% TPO-type photoinitiator: 1–<5%	Saremco Dental AG, Rebstein, Switzerland

**Table 2:** Descriptive statistics of fracture load values (N) for lithium disilicate (LDC) and 3D-printed resin (3D) molar crowns with different occlusal thicknesses (0.5, 1.0 and 1.5 mm) after thermomechanical aging.

Group (*n* = 30)	Thickness (*n* = 10)	Mean (N)	SD	Minimum Value	Maximum Value	95% Confidence Interval for Mean	
						Lower Bound	Upper Bound
3D	0.5 mm	451	98	312	596	381	521
	1.0 mm	1214	187	940	1548	1080	1348
	1.5 mm	1365	232	1005	1708	1199	1531
LDC	0.5 mm	542	113	358	741	461	623
	1.0 mm	1634	289	1218	2019	1427	1841
	1.5 mm	1802	318	1456	2438	1575	2029

SD: Standard deviation.

**Table 3:** Two-way ANOVA results showing the effects of restorative material type (3D-LDC), occlusal thickness (0.5, 1.0, and 1.5 mm), and their interaction on fracture resistance.

Fracture Resistance	df	F	*p* Value	ηp^2^
Material	1	82.6	<0.001	0.60
Thickness	2	109.4	<0.001	0.80
Material * Thickness	2	7.2	<0.001	0.21
Error	54	-	-	-

Degrees of freedom (df) are shown for each effect, and partial ηp^2^ indicates the effect size. * Interaction between material and thickness.

**Table 4:** Tukey’s HSD pairwise comparisons of fracture resistance values between lithium disilicate (LDC) and 3D-printed resin (3D) crowns at each occlusal thickness level.

Thickness	Material	Mean Difference (N)	Std Error	*p* Value	95%CI for Difference	
					Lower	Upper
0.5 mm	LDC-3D	91	69.8	0.048 *	−49	231
1.0 mm	LDC-3D	420	69.8	0.003 *	280	560
1.5 mm	LDC-3D	437	69.8	0.028 *	297	577

* Significant difference.

**Table 5:** Percentage change and 95% confidence intervals (CI) for the mean differences in fracture resistance (N) between occlusal thickness groups (0.5, 1.0 and 1.5 mm) for 3D-printed resin (3D) and lithium disilicate (LDC) crowns.

Group (*n* = 30)	Thickness (A)	Thickness (B)	% Change (B − A)	Sig	95%CI for Difference	
					Lower	Upper
3D	0.5 mm	1.0 mm	169.18	*	692	834
	0.5 mm	1.5 mm	202.66	*	826	1002
	1.0 mm	1.5 mm	12.44	ns	−22	324
LDC	0.5 mm	1.0 mm	201.48	*	1005	1179
	0.5 mm	1.5 mm	232.47	*	1155	1365
	1.0 mm	1.5 mm	10.28	ns	−26	362

“*” indicates a statistically significant difference (*p* < 0.05); “ns” = non-significant. The percentage change values were provided for descriptive purposes only.

**Table 6:** Distribution of fracture patterns (repairable, non-repairable) for lithium disilicate (LDC) and 3D-printed resin (3D) crowns with different occlusal thicknesses (0.5, 1.0 and 1.5 mm).

Thickness	Test Group	
	3D	LDC
0.5 mm	0 R/10 NR	0 R/10 NR
1.0 mm	7 R/3 NR	5 R/5 NR
1.5 mm	9 R/1 NR	7 R/3 NR

R: repairable, NR: non-repairable.

## Data Availability

The original contributions presented in this study are included in the article. Further inquiries can be directed to the corresponding author.

## Abbreviations

The following abbreviations are used in this manuscript:

3D	Three-dimensional
ANOVA	Analysis of variance
BisEMA	Bisphenol A polyethylene glycol diether dimethacrylate
CAD	Computer-aided design
CAM	Computer-aided manufacturing
CI	Confidence interval
HF	Hydrofluoric acid
HSD	Honestly significant difference
LDC	Lithium disilicate ceramic
NR	Non-repairable failure
PDL	Periodontal ligament
R	Repairable failure
SD	Standard deviation
SEM	Scanning electron microscopy
SLA	Stereolithography

## References

[1] Tian Y., Chen C., Xu X., Wang J., Hou X., Li K., Lu X., Shi H., Lee E.S., Jiang H.B. (2021). A Review of 3D Printing in Dentistry: Technologies, Affecting Factors, and Applications. Scanning.

[2] Małysa A., Jenčová J., Weżgowiec J. (2025). Biocompatibility of CAD/CAM Milled Dental Restorative Materials: A Systematic Review from In Vitro Studies. Materials.

[3] Baba K. (2021). Database-Driven Prosthodontics-Future of Digital Dentistry. J. Prosthodont. Res..

[4] Alharbi A., Ardu S., Bortolotto T., Krejci I. (2017). Stain susceptibility of composite and ceramic CAD/CAM blocks versus direct resin composites with different resinous matrices. Odontology.

[5] Donmez M.B., Olcay E.O., Demirel M. (2022). Load-to-Failure Resistance and Optical Characteristics of Nano-Lithium Disilicate Ceramic after Different Aging Processes. Materials.

[6] Berger L., Förtsch F., Kretschmer R.R., Sednyev O., Zorzin J.I., Wichmann M., Matta R.E. (2025). Influence of the Milling Strategy on the Marginal Fit of Chairside-Fabricated Lithium Disilicate Crowns. Materials.

[7] Ma L., Guess P.C., Zhang Y. (2013). Load-bearing properties of minimal-invasive monolithic lithium disilicate and zirconia occlusal onlays: Finite element and theoretical analyses. Dent. Mater..

[8] Lawson N.C., Bansal R., Burgess J.O. (2016). Wear, strength, modulus and hardness of CAD/CAM restorative materials. Dent. Mater..

[9] Rekow E.D., Silva N.R., Coelho P.G., Zhang Y., Guess P., Thompson V.P. (2011). Performance of dental ceramics: Challenges for improvements. J. Dent. Res..

[10] Sasse M., Krummel A., Klosa K., Kern M. (2015). Influence of restoration thickness and dental bonding surface on the fracture resistance of full-coverage occlusal veneers made from lithium disilicate ceramic. Dent. Mater..

[11] Daher R., Ardu S., di Bella E., Krejci I., Duc O. (2024). Efficiency of 3D printed composite resin restorations compared with subtractive materials: Evaluation of fatigue behavior, cost, and time of production. J. Prosthet. Dent..

[12] Rezaie F., Farshbaf M., Dahri M., Masjedi M., Maleki R., Amini F., Wirth J., Moharamzadeh K., Weber F.E., Tayebi L. (2023). 3D Printing of Dental Prostheses: Current and Emerging Applications. J. Compos. Sci..

[13] Pot G.J., Van Overschelde P.A., Keulemans F., Kleverlaan C.J., Tribst J.P.M. (2024). Mechanical Properties of Additive-Manufactured Composite-Based Resins for Permanent Indirect Restorations: A Scoping Review. Materials.

[14] Zimmermann M., Ender A., Egli G., Özcan M., Mehl A. (2019). Fracture load of CAD/CAM-fabricated and 3D-printed composite crowns as a function of material thickness. Clin. Oral Investig..

[15] Alamoush R.A., Silikas N., Salim N.A., Al-Nasrawi S., Satterthwaite J.D. (2018). Effect of the Composition of CAD/CAM Composite Blocks on Mechanical Properties. Biomed. Res. Int..

[16] Tahayeri A., Morgan M., Fugolin A.P., Bompolaki D., Athirasala A., Pfeifer C.S., Ferracane J.L., Bertassoni L.E. (2018). 3D printed versus conventionally cured provisional crown and bridge dental materials. Dent. Mater..

[17] Della Bona A., Cantelli V., Britto V.T., Collares K.F., Stansbury J.W. (2021). 3D printing restorative materials using a stereolithographic technique: A systematic review. Dent. Mater..

[18] Suksuphan P., Krajangta N., Didron P.P., Wasanapiarnpong T., Rakmanee T. (2024). Marginal adaptation and fracture resistance of milled and 3D-printed CAD/CAM hybrid dental crown materials with various occlusal thicknesses. J. Prosthodont. Res..

[19] Magne P., Schlichting L.H., Maia H.P., Baratieri L.N. (2010). In vitro fatigue resistance of CAD/CAM composite resin and ceramic posterior occlusal veneers. J. Prosthet. Dent..

[20] Valenzuela E.B.S., Andrade J.P., da Cunha P., Bittencourt H.R., Spohr A.M. (2021). Fracture load of CAD/CAM ultrathin occlusal veneers luted to enamel or dentin. J. Esthet. Restor. Dent..

[21] Egbert J.S., Johnson A.C., Tantbirojn D., Versluis A. (2015). Fracture strength of ultrathin occlusal veneer restorations made from CAD/CAM composite or hybrid ceramic materials. Oral Sci. Int..

[22] Sorrentino R., Triulzio C., Tricarico M.G., Bonadeo G., Gherlone E.F., Ferrari M. (2016). In vitro analysis of the fracture resistance of CAD-CAM monolithic zirconia molar crowns with different occlusal thickness. J. Mech. Behav. Biomed. Mater..

[23] Bolaca A., Erdoğan Y. (2024). Fracture resistance evaluation of CAD/CAM zirconia and composite primary molar crowns with different occlusal thicknesses. J. Appl. Biomater. Funct. Mater..

[24] Yılmaz G., Yeşil Z. (2025). A new era in provisional restorations: Evaluating marginal accuracy and fracture strength in additive, substractive and conventional techniques. BMC Oral Health.

[25] Nordahl N., Vult von Steyern P., Larsson C. (2015). Fracture strength of ceramic monolithic crown systems of different thickness. J. Oral Sci..

[26] Potiket N., Chiche G., Finger I.M. (2004). In vitro fracture strength of teeth restored with different all-ceramic crown systems. J. Prosthet. Dent..

[27] May L.G., Kelly J.R., Bottino M.A., Hill T. (2012). Effects of cement thickness and bonding on the failure loads of CAD/CAM ceramic crowns: Multi-physics FEA modeling and monotonic testing. Dent. Mater..

[28] Dauti R., Lilaj B., Heimel P., Moritz A., Schedle A., Cvikl B. (2020). Influence of two different cement space settings and three different cement types on the fit of polymer-infiltrated ceramic network material crowns manufactured using a complete digital workflow. Clin. Oral Investig..

[29] Tartuk B.K., Akın Tartuk G. (2026). Impact of Thermomechanical Aging on Marginal Fit and Fracture Resistance of CAD/CAM Endocrowns Fabricated from Different Materials. Polymers.

[30] Song M.G., Ko K.H., Huh Y.H., Park C.J., Cho L.R. (2025). Edge Chipping Resistance and Flexural Strength of CAD-CAM Ceramics Before and After Thermomechanical Aging. J. Esthet. Restor. Dent..

[31] Rosentritt M., Sawaljanow A., Behr M., Kolbeck C., Preis V. (2015). Effect of tooth brush abrasion and thermo-mechanical loading on direct and indirect veneer restorations. Clin. Oral Investig..

[32] Ruse N.D., Sadoun M.J. (2014). Resin-composite blocks for dental CAD/CAM applications. J. Dent. Res..

[33] Amesti-Garaizabal A., Agustín-Panadero R., Verdejo-Solá B., Fons-Font A., Fernández-Estevan L., Montiel-Company J., Solá-Ruíz M.F. (2019). Fracture Resistance of Partial Indirect Restorations Made with CAD/CAM Technology. A Systematic Review and Meta-analysis. J. Clin. Med..

[34] Park Y., Kim J., Kang Y.J., Shim E.Y., Kim J.H. (2024). Comparison of Fracture Strength of Milled and 3D-Printed Crown Materials According to Occlusal Thickness. Materials.

[35] Abd ElAziz R.H., Ragab R.A., Elzayat G.A. (2024). Comparative evaluation and patient satisfaction with an electrical impedance-based device versus digital radiography in the estimation of remaining dentin thickness in carious posterior permanent teeth: (Diagnostic accuracy study). BMC Oral Health.

[36] Schlichting L.H., Maia H.P., Baratieri L.N., Magne P. (2011). Novel-design ultra-thin CAD/CAM composite resin and ceramic occlusal veneers for the treatment of severe dental erosion. J. Prosthet. Dent..

[37] Bergamo E.T.P., Bordin D., Ramalho I.S., Lopes A.C.O., Gomes R.S., Kaizer M., Witek L., Bonfante E.A., Coelho P.G., Del Bel Cury A.A. (2019). Zirconia-reinforced lithium silicate crowns: Effect of thickness on survival and failure mode. Dent. Mater..

[38] Corbani K., Hardan L., Skienhe H., Özcan M., Alharbi N., Salameh Z. (2020). Effect of material thickness on the fracture resistance and failure pattern of 3D-printed composite crowns. Int. J. Comput. Dent..

[39] Charoenporn W., Sornsuwan T., Sae-Lee D., Amornvit P., Chaijareenont P., Rungsiyakull P. (2024). Evaluating fatigue resistance in occlusal veneers: A comparative study of processing techniques and material thickness of lithium disilicate (IPS e.max Press vs. IPS e.max CAD). BMC Oral Health.

[40] Elian El Hayek J., El Osta N., Farhat Mchayleh N. (2022). Fracture strength of preformed zirconia crown and new custom-made zirconia crown for the restoration of deciduous molars: In vitro study. Eur. Arch. Paediatr. Dent..

[41] Refaie A., Bourauel C., Fouda A.M., Keilig L., Singer L. (2023). The effect of cyclic loading on the fracture resistance of 3D-printed and CAD/CAM milled zirconia crowns-an in vitro study. Clin. Oral Investig..

[42] Ladino L., Sanjuan M.E., Valdez D.J., Eslava R.A. (2021). Clinical and Biomechanical Performance of Occlusal Veneers: A Scoping Review. J. Contemp. Dent. Pract..

[43] Essam N., Soltan H., Attia A. (2023). Influence of thickness and surface conditioning on fracture resistance of occlusal veneer. BMC Oral Health.

[44] Monteiro J.B., Riquieri H., Prochnow C., Guilardi L.F., Pereira G.K.R., Borges A.L.S., de Melo R.M., Valandro L.F. (2018). Fatigue failure load of two resin-bonded zirconia-reinforced lithium silicate glass-ceramics: Effect of ceramic thickness. Dent. Mater..

[45] Szczesio-Wlodarczyk A., Domarecka M., Kopacz K., Sokolowski J., Bociong K. (2021). An Evaluation of the Properties of Urethane Dimethacrylate-Based Dental Resins. Materials.

[46] Pratap B., Gupta R.K., Bhardwaj B., Nag M. (2019). Resin based restorative dental materials: Characteristics and future perspectives. Jpn. Dent. Sci. Rev..

[47] Edelhoff D., Sorensen J.A. (2002). Tooth structure removal associated with various preparation designs for anterior teeth. J. Prosthet. Dent..

[48] Zahran M., Abo El-Farag S., Soltan H., Attia A. (2023). Fracture load of ultrathin occlusal veneers: Effect of thickness and surface conditioning. J. Mech. Behav. Biomed. Mater..

[49] Aslam A., Hassan S., Nayyer M., Ahmed B. (2018). Intraoral repair protocols for fractured metal-ceramic restorations-Literature review. S. Afr. Dent. J..

[50] Macura A., Kasperski J., Urbaniak M., Klimek A. (2013). Materials and methods used for repairing damaged veneering porcelain in permanent complex prosthetic restorations—Review of literature. J. Stomatol..

[51] Di Fiore A., Stellini E., Alageel O., Alhotan A. (2024). Comparison of mechanical and surface properties of two 3D printed composite resins for definitive restoration. J. Prosthet. Dent..

[52] Xiao S., Zhang R.J., Tan F.B. (2025). Effect of thermal cycling on the mechanical properties of conventional, milled, and 3D-printed base resin materials: A comparative in vitro study. PeerJ.

[53] El-Damanhoury H.M., Haj-Ali R.N., Platt J.A. (2015). Fracture resistance and microleakage of endocrowns utilizing three CAD-CAM blocks. Oper. Dent..

[54] Albelasy E., Hamama H.H., Tsoi J.K.H., Mahmoud S.H. (2021). Influence of material type, thickness and storage on fracture resistance of CAD/CAM occlusal veneers. J. Mech. Behav. Biomed. Mater..

[55] Ko S., Davey J., Douglass S., Yang J., Tuttle M.E., Salviato M. (2019). Effect of the thickness on the fracturing behavior of discontinuous fiber composite structures. Compos. Part A Appl. Sci. Manuf..

[56] Baldissara P., Monaco C., Onofri E., Fonseca R.G., Ciocca L. (2019). Fatigue resistance of monolithic lithium disilicate occlusal veneers: A pilot study. Odontology.

[57] González-Gil D., Flores-Fraile J., Vera-Rodríguez V., Martín-Vacas A., López-Marcos J. (2024). Comparative Meta-Analysis of Minimally Invasive and Conventional Approaches for Caries Removal in Permanent Dentition. Medicina.

[58] Anusavice K.J., Kakar K., Ferree N. (2007). Which mechanical and physical testing methods are relevant for predicting the clinical performance of ceramic-based dental prostheses?. Clin. Oral Implant. Res..

